# Health-Care Delay in Malignant Melanoma: Various Pathways to Diagnosis and Treatment

**DOI:** 10.1155/2014/294287

**Published:** 2014-01-05

**Authors:** Senada Hajdarevic, Åsa Hörnsten, Elisabet Sundbom, Ulf Isaksson, Marcus Schmitt-Egenolf

**Affiliations:** ^1^Department of Nursing, Umeå University, Umeå, Sweden; ^2^Department of Clinical Sciences, Division of Psychiatry and Medical Psychology, Umeå University, Umeå, Sweden; ^3^Department of Public Health and Clinical Medicine, Dermatology and Venereology, Umeå University, Umeå, Sweden

## Abstract

We aimed to describe and compare patients diagnosed with malignant melanoma (MM), depending on their initial contact with care and with regard to age, sex, and MM type and thickness, and to explore pathways and time intervals (lead times) between clinics from the initial contact to diagnosis and treatment. The sample from northern Sweden was identified via the Swedish melanoma register. Data regarding pathways in health care were retrieved from patient records. In our unselected population of 71 people diagnosed with skin melanoma of SSM and NM types, 75% of patients were primarily treated by primary health-care centres (PHCs). The time interval (delay) from primary excision until registration of the histopathological assessment in the medical records was significantly longer in PHCs than in hospital-based and dermatological clinics (Derm). Thicker tumors were more common in the PHC group. Older patients waited longer times for wide excision. Most MM are excised rapidly at PHCs, but some patients may not be diagnosed and treated in time. Delay of registration of results from histopathological assessments within PHCs seems to be an important issue for future improvement. Exploring shortcomings in MM patients' clinical pathways is important to improve the quality of care and patient safety.

## 1. Introduction

Malignant melanoma (MM) incidence is increasing globally, and Sweden is among the top 10 countries in the world with regard to incidence [1]. During the last decade, MM has become the sixth most common form of cancer in Sweden [2]. MM is a skin cancer with fatal outcome, if not diagnosed and treated in time [3]. A critical point in the development of MM is the penetration of the dermal-epidermal basement membrane, which highly increases the risk for metastases [4]. The optimal way to cure MM is therefore early detection and excision. The reduction of both patient and doctor's delay is of key importance for early diagnosis and clinical outcome of MM.

One reason for delayed diagnosis relates to patients' care-seeking patterns for suspected MM [5]. A review of the literature concerning patient delay highlights health beliefs, low sense of severity, and susceptibility related to melanoma as reasons for delayed care seeking. Other reasons are related to gender, age, and living conditions [5–7].

In the health-care organization, reasons for late diagnosis of cancer in general have been related to accessibility, difficulties and complexity in procedures of diagnosis and incorrect referrals [8–12]. Despite its importance, reasons for health-care and doctor's delay in MM have been only sparsely investigated. Earlier studies emphasized difficulties in diagnostics [5, 13], as well as low access to general practitioners (GPs) [14], and gatekeeping [11]. Baade et al. [13] have described the diagnostic process and highlighted the important role of GPs and the emerging role of primary care skin clinics. They also reported that older people from rural areas needed special attention and intervention since both patient delay and health-care delay are prolonged.

The need for quality assurance in the health-care system has become generally appreciated. Analysis of clinical pathways and lead times can detect opportunities for improvement. Murchie et al. [14] compared melanoma health-care delay during the diagnostic pathways in the United Kingdom, Sweden, and the Netherlands and found differences in time delay in secondary care between countries, in which Scotland had the highest delay.

Guidelines for treatment of MM [3, 15] are important for patients' clinical pathways to know how to act and, if needed, where to refer patients with suspected MM. Since prognosis is strongly related to tumor thickness [16], timely treatment is essential for optimal outcomes. In some guidelines, time limits for referrals or excision of suspected melanomas are pronounced, where the primary excision of a suspected lesion should be done within two weeks [3, 17]. European consensus declares that the definitive surgical excision should be performed with wide safety margins, preferably within 4–6 weeks after initial diagnosis [3]. Swedish guidelines omit such recommendations [15, 18].

Only a few studies have described pathways and lead times between clinics from initial care seeking to diagnosis and treatment for MM [13, 14, 19]. The aim of this study was to describe and compare patients diagnosed with MM, depending on their initial contact with care, with regard to age, sex, and MM type and thickness, and to explore pathways and lead times between clinics from the initial contact to diagnosis and treatment.

## 2. Materials and Methods

### 2.1. Participants

The melanoma register identified 134 people meeting the inclusion criteria: aged 18–80 years and diagnosed with skin melanoma—superficial spreading melanoma (SSM), nodular melanoma (NM), or melanoma *in situ*—between January 2008 and December 2010. Less frequent subtypes such as ALM (acral lentiginous melanoma) and LMM (lentigo maligna melanoma) were excluded, since they have a differing biological behaviour.

Completing data about the clinical pathways were collected from the computerized patient records. Deceased people (*n* = 5), those who had moved to other counties (*n* = 2), people with documented severe mental illness (*n* = 1), and those diagnosed with melanoma more than once (*n* = 3) were excluded. In all, 123 people were asked to participate. Among those, 35 declined, 17 did not respond after two reminders, and 71 (58%) participants gave informed consent and were included in the study. Characteristics of the participants are given in Table 1.

The participants were divided into two groups, depending on where they initially sought care. The first group were patients who were recruited from the public primary health-care centers (PHCs) and the second group from dermatological clinics at hospitals, other hospital specialist clinics, or private skin clinics (Derm). Data were analyzed following the clinical pathways and lead times for each patient as documented in their medical records. The elapsed time between the milestones in the pathway was analyzed and compared between groups of patients, based on age, sex, and MM characteristics.

### 2.2. Data Collection

During the spring of 2011, we collected data from the National Quality Register for Melanoma of the Skin of the northern Swedish region and patients' medical records from the County of Västerbotten in northern Sweden. The data collection consisted of dates for important events (milestones) between clinics and examinations in accordance with the regional guidelines for MM [18] (Tables 2(a) and 2(b)). Data regarding tumor thickness, histogenetic subtype of melanoma, registered result from first histopathological diagnosis, and the reporting clinics were collected from the register.

### 2.3. Statistical Analysis

Descriptive statistics were used to describe the background data. In order to explore differences in lead times between patients seeking care at either a PHC or Derm, the pathway was divided into important milestones (Tables 2(a) and 2(b)). The Chi-square test was used for dichotomous data and Mann-Whitney *U* test for continuous data to compare differences between the groups due to skewness in distribution of data. A *P* value <0.05 was chosen as the level for significance in all tests. For all analyses, SPSS, ver. 18.0 was used.

### 2.4. Ethics

The study obtained approval from the Regional Ethics Review Board in Umeå (Dnr 2011-88-32). Before sending invitations and reminders to patients, we updated information about deceased persons from the Swedish Population Register, with the intention of sparing the relatives unnecessary distress.

## 3. Results

The results showed that 53 (75%) patients had initially sought care and were primarily treated for suspected MM at PHCs (Table 1). The remaining 18 (25%) patients sought care at other clinics (Derm), that is, the public dermatological hospital clinic, 11 (15.4%); other hospital clinics, 4 (5.6%); and private skin clinics, 3 (4%).

From the physician's assessment to primary excision, 38 (72%) patients were managed within their own clinics in the PHC group compared to 12 (67%) in the Derm group (*P* = ns). Patients whose lesions had not been excised within their own clinics were referred to surgery clinics for primary excision. In total, 24 patients were referred once, and 7 patients were referred twice before primary excision. Ten percent (*n* = 7) of all patients underwent a biopsy before the primary excision. After receiving results from the histopathological diagnosis, 36 (95%) patients from the PHC group were referred to surgical clinics for wide excision compared to eight (40%) patients from the Derm group who were referred to another clinic for wide excision. A wide excision, that is, a margin of 5–20 mm, depending on the initial Breslow thickness, was performed on 67 (94%) patients, 87% at surgical clinics (*n* = 58) and 13% at dermatological clinics (*n* = 9). The remaining patients (6%) were diagnosed within dermatological clinics as having *in situ* MM and were followed up there. Sixty-four patients (91%) were followed up after treatment. Four percent were assessed as not in need of any follow-up. Among those, two participants had *in situ* melanoma, and one had SSM 0.60 mm. For the remaining 5%, information was lacking in the patient records.

The results showed that PHCs primarily treated patients with more severe types of MM (Table 1). Furthermore (not presented in tables), in the PHC group, *in situ* MM was more common among women than among men (86.7% versus 13.3%, *P* = 0.020). There were no significant differences in age or sex between patients of the PHC and Derm groups (Table 3).

The time from first physician's assessment to the preexcision referral was significantly higher and almost doubled in the PHC group compared to the Derm group (35 versus 20 days, *P* = 0.024) (Tables 2(a) and 2(b)).

The range from the physician's assessment to primary excision was wide in both groups (0–131 and 0–67 days, resp.); however, no significant differences between groups were found.

Significant differences in time interval (delay) were found between the PHC and Derm groups from primary excision until registration of the histopathological diagnosis in the medical records. The delay was significantly longer at PHCs (13 versus 6.5 days, *P* = 0.001) (Tables 2(a) and 2(b)).

One result (not presented in tables) showed that people with thicker melanomas (>0.70 mm) waited significantly longer to be referred for follow-up than those with thinner MM (10 versus 0 days, *P* = 0.001). People older than 60 years waited significantly longer from first histopathological diagnosis to wide excision than younger patients (38 versus 28 days, *P* = 0.005) and also from referral for wide excision to wide excision (35 versus 21.5 days, *P* = 0.029).

We found that women waited a shorter time from the first physician's assessment to the primary excision compared to men (0 versus 18 days, *P* = 0.052) and also waited a shorter time from referral for wide excision to wide excision (21 versus 35.5 days, *P* = 0.031). In addition, women had a tendency towards a shorter waiting time from first physician's assessment to follow-up, compared to men (108.5 versus 150 days, *P* = 0.059).

## 4. Discussion

We found differences in health-care pathways and lead times between groups, depending on where people started to seek care. The time from primary excision until the result of the histopathological diagnosis recorded in the medical records was nearly twice as long for those who were seeking care at PHCs as for those who were seeking care at hospital or dermatological clinics. More precisely, it differed by 6.5 days just for registration, which is not optimal among patients with an aggressive cancer such as MM, since the histopathological diagnosis is a crucial moment for a physician to decide upon further treatment [3]. This delay, documented in medical patient records, is consistent with a national report [20] that revealed long waiting times from primary excision until patients received information about the diagnosis. The report presented differences of 4.3 times (in days) between the lowest and the highest median waiting time.

It is difficult to analyze the reasons for this difference, due to the complexity of the administrative health-care system. Our investigation is based on registration dates in patient records. One explanation might be that PHCs professionals are overloaded [21], which could account for a delayed document registration in the medical patient record. The number of patient visits at PHCs has increased 10% during 2005–2009, while specialist care visits only increased by 2%. We also found that the median of time from the first physician's assessment to primary excision was short, independent of initial contact clinic, which is encouraging. However, the ranges within both groups were unfortunately wide (Table 2(b)). Although such results are difficult to interpret, it is important to present them, in order to identify obstacles in the clinical pathways for patients with malignant melanoma, and thereby improve patient safety. Organizational problems during vacation periods, or incorrect referrals or misconceptions between clinics could also contribute to such delay [20].

We also found that patients who sought care at PHCs had more severe and thicker melanoma than patients treated at hospital and dermatological clinics (Table 3). Melanoma, in general and particularly NM, is more common among older people [22], who traditionally more often seek care at PHCs. This may explain why thicker melanoma is more common there. Older patients in our study waited a longer time for wide excision. The National Board of Health and Welfare [20] has recently reported that older people with cancer in general wait longer for appointments with physicians and for care, which we also found. Accessibility, lack of information, and long wait times to diagnosis are common problems within health care, particularly within cancer care [23]. Nurses could preferably act as coordinators to speed up the process of diagnosis and treatment.

Furthermore, we observed that about 10% of all participants underwent biopsies before primary excision, which is not in line with guidelines [3, 15]. This implies that physicians do not suspect some of those lesions as MM, and thereby contribute to a delayed diagnosis [24].

Women's shorter health-care delay regarding primary excision and referral for wide excision to wide excision can be related to their thinner tumors and better prognosis. Since women's care-seeking delay is shorter and they more often detect MM by themselves than men do [5, 7], they may request quicker further treatment. The highest delay in both PHC and Derm groups concerned the time from the referral for wide excision to the wide excision, which in median was 50.0 versus 57.5 days and thereby something that certainly could be improved (Table 2(b)).

## 5. Methodological Discussion

The total local population of all people 18–80 years diagnosed with SSM, NM, and *in situ* MM during the past 3 years was identified by the melanoma register and invited to participate. The Swedish law requires informed consent for this kind of study. Unfortunately, we were only able to achieve a 58% rate of acceptance. However, the sample concurs with the distribution of melanoma in the area of the study, which indicates a representative sample. Furthermore, a missing-case analysis showed no significant differences between participants and nonparticipants concerning gender (male gender 46.5% versus 42.3%, *P* = 0.646), mean age (57.92 years versus 58.00 years, *P* = 0.973), mean tumor thickness (1.02 mm versus 1.16 mm, *P* = 0.602), or type of melanoma (*in situ* 31% versus 25%; SSM 53.5% versus 57.3%; NM 15.5% versus 17.3%, *P* = 0.766). However, we cannot totally exclude the possibility that the missing data may affect the results.

The reliability of the documentation of the first contact with the health-care service is a limitation of using data from patients' records. Records show that most patients had their melanomas excised at day one. However, we estimate that many patients had contacted a nurse or physician by telephone to get an appointment time at least 1–7 days before the first visit, sometimes longer. Nevertheless, if not registered in the record, we cannot verify if and/or when such a precontact was made.

## 6. Conclusions

PHCs were, during the period of data collection, the primary contact clinic for MM patients in this region of Northern Sweden. Most MMs are excised rapidly, but for some patients the time for diagnosis and treatment may have been prolonged. Delay from primary excision until registration of the results from histopathological diagnosis within PHCs seems to be an important issue for future improvement. Exploring delay in MM patients' clinical pathways is important for improving the quality of care and patient safety. To reduce total delay of treatment in MM, future studies should focus on the time interval between first discovery of a suspect lesion through final treatment, since patient delay far exceeds health-care delay.

## Figures and Tables

**Table 1:** Characteristics of included participants: gender related to age, tumor type, tumor thickness, and initial contact clinic.

Total	All (*n* = 71)	Women (*n* = 38)	Men (*n* = 33)	*P*-value
Age (yrs)				
Median	60.0	56.5	63.0	0.059^1^
Range	30–80	30–79	32–80	
≤60 (*n* (%))	38 (53.5%)	24 (63.2%)	14 (42.4%)	0.081^2^
>60 (*n* (%))	33 (46.5%)	14 (36.8%)	19 (57.6%)	
Tumor type (*n* (%))				
In situ	22 (31.0%)	18 (47.0%)	4 (12.0%)	0.006^2^
SSM	38 (53.5%)	15 (40.0%)	23 (70.0%)	
NM	11 (15.5%)	5 (13.0%)	6 (18.0%)	
Tumor thickness (mm)				
Mean (SD)	1.01 (1.19)	0.84 (1.26)	1.21 (1.09)	
Median (range)	0.75 (0.00–5.90)	0.25 (0.00–5.90)	0.90 (0.00–4.10)	0.023^1^
≤0.70 (*n* (%))	35 (49.3%)	23 (60.5%)	12 (36.4%)	0.042^2^
>0.70 (*n* (%))	36 (50.7%)	15 (39.5%)	21 (63.6%)	
Initial contact clinic (*n* (%))				
PHCs	53 (74.6%)	30 (78.9%)	23 (69.7%)	0.372^2^
Derm	18 (25.4%)	8 (21.1%)	10 (30.3%)	

^1^Mann-Whitney *U* test. ^2^Chi-square test. SSM: superficial spreading melanoma, NM: nodular melanoma, PHCs: primary health-care centres, Derm: dermatological and other specialist clinics.

**Table:** (a)

Milestones	Event marked by milestone
0 = initial contact	Patient booked the first appointment
A = assessment by physician	The first assessment by a physician
B = preexcision referral	Referral for primary excision
C = primary excision	Primary excision
D = histopathological diagnosis I	Result from first histopathological diagnosis
E = referral for wide excision	Referral for wide excision
F = wide excision	Wide excision surgery
G = histopathological diagnosis II	Result from second histopathological diagnosis
H = follow-up referral	Referral for follow-up
I = follow-up	Follow-up visit

**Table:** (b)

Milestone transition	PHCs (days)			Derm (days)				Participants (*n*)
	Mean	Median	Range	Mean	Median	Range	P*	
A → B	11.8	0	0–98	3.0	0	0–13	ns	24
B → C	41.6	35.0	13–131	19.0	20.0	5–34	0.024	24
A → C	19.9	0	0–131	14.1	4.5	0–67	ns	71
C → D	16.0	13.0	1–134	7.2	6.5	1–17	0.001	71
D → E	7.2	5.0	0–28	22.1	4.0	0–121	ns	53
E → F	74.4	50.0	20–374	121.5	57.5	15–528	ns	52
F → G	13.2	12.0	0–64	9.3	8.0	1–25	ns	65
G → H	10.6	7.0	0–60	11.9	2.0	0–63	ns	44**
H → I	63.4	42.5	1–353	54.3	43.0	4–148	ns	43**

*Mann-Whitney *U* test.

**Seven patients excluded, as referral was sent before registration of histopathology diagnosis II.

**Table 3:** Comparison between primary health-care centers (PHC) and dermatological and other clinic groups (Derm) as related to age, sex, tumor thickness, and type.

	Total	PHCs	Derm	*P*-value
Age (yrs)				
Median	60	58	60.5	0.726^1^
≤60 (*n* (%))	38 (53.5)	29 (54.7)	9 (50.0)	0.729^2^
Sex				
Women (*n* (%))	53 (74.6)	30 (56.6)	8 (44.4)	0.372^2^
Men (*n* (%))	18 (25.4)	23 (43.4)	10 (56.6)	
Tumor thickness (mm)				
Mean/median	1.01/0.75	1.12/0.80	0.70/0.37	0.140^1^
>0.70 (*n* (%))	36 (50.7)	29 (54.7)	7 (38.9)	0.246^2^
Tumor type (*n* (%))				
SSM	38 (53.5)	28 (52.8)	10 (55.6)	0.360^2^
NM	11 (15.5)	10 (18.9)	1 (5.6)	
In situ	22 (31.0)	15 (28.3)	7 (38.9)	

^1^Mann-Whitney *U* test. ^2^Chi-square test. SSM: superficial spreading melanoma, NM: nodular melanoma, PHCs: primary health-care centers, Derm: dermatological and other clinics.
